# Histological characteristics of Acute Tubular Injury during Delayed Graft Function predict renal function after renal transplantation

**DOI:** 10.14814/phy2.14000

**Published:** 2019-02-28

**Authors:** Tobias T. Pieters, Lucas L. Falke, Tri Q. Nguyen, Marianne C. Verhaar, Sandrine Florquin, Frederike J. Bemelman, Jesper Kers, Thomas Vanhove, Dirk Kuypers, Roel Goldschmeding, Maarten B. Rookmaaker

**Affiliations:** ^1^ Department of Nephrology and Hypertension University Medical Center Utrecht Utrecht The Netherlands; ^2^ Department of Pathology University Medical Center Utrecht Utrecht The Netherlands; ^3^ Department of Internal Medicine Diakonessenhuis Utrecht The Netherlands; ^4^ Department of Pathology Amsterdam University Medical Centers Amsterdam The Netherlands; ^5^ Department of Nephrology Amsterdam University Medical Centers Amsterdam The Netherlands; ^6^ University of Amsterdam Van ‘t Hoff Institute for Molecular Sciences (HIMS) Amsterdam The Netherlands; ^7^ Department of Nephrology University Hospitals of Leuven Leuven Belgium

**Keywords:** ATI, CD133, delayed graft function, histology, Ki67, proliferation, regeneration, renal transplantation

## Abstract

Acute Tubular Injury (ATI) is the leading cause of Delayed Graft Function (DGF) after renal transplantation (RTX). Biopsies taken 1 week after RTX often show extensive tubular damage, which in most cases resolves due to the high regenerative capacity of the kidney. Not much is known about the relation between histological parameters of renal damage and regeneration immediately after RTX and renal outcome in patients with DGF. We retrospectively evaluated 94 patients with DGF due to ATI only. Biopsies were scored for morphological characteristics of renal damage (edema, casts, vacuolization, and dilatation) by three independent blinded observers. The regenerative potential was quantified by tubular cells expressing markers of proliferation (Ki67) and dedifferentiation (CD133). Parameters were related to renal function after recovery (CKD‐EPI 3, 6, and 12 months posttransplantation). Quantification of morphological characteristics was reproducible among observers (Kendall's W ≥ 0.56). In a linear mixed model, edema and casts significantly associated with eGFR within the first year independently of clinical characteristics. Combined with donor age, edema and casts outperformed the Nyberg score, a well–validated clinical score to predict eGFR within the first year after transplantation (*R* ^2^ = 0.29 vs. *R* ^2^ = 0.14). Although the number of Ki67+ cells correlated to the extent of acute damage, neither CD133 nor Ki67 correlated with renal functional recovery. In conclusion, the morphological characteristics of ATI immediately after RTX correlate with graft function after DGF. Despite the crucial role of regeneration in recovery after ATI, we did not find a correlation between dedifferentiation marker CD133 or proliferation marker Ki67 and renal recovery after DGF.

## Introduction

Acute tubular injury (ATI) is a common complication following renal transplantation (RTX), causing short–term graft failure called Delayed Graft Function (DGF), during which patients require dialysis. ATI during DGF, once subsided, yields an increased risk of gradual histological and functional decline (Yarlagadda et al. 2009; Gosset et al. 2017). Renal recovery after DGF is determined by the balance between inflicted damage and ensuing regeneration. Although the mechanisms underlying ATI and subsequent regeneration are progressively identified (Ferenbach and Bonventre 2015), predicting outcome after ATI remains challenging and currently depends mainly on clinical characteristics.

ATI is characterized by specific histological changes of the tubules. After loss of brush border and nonisometric cytoplasmic vacuolization, tubular cells undergo cytoplasmic fragmentation or detach entirely into the lumen, producing obstructive casts (Nonclercq et al. 1989; Solez et al. 1993). In parallel, tubular dilatation is seen due to increased intraluminal pressure and alterations in the cytoskeleton (Shimizu et al. 1994). Because of obstruction and inflammation, fluid oozes through the denuded basal membrane causing interstitial edema. Signs of apoptosis and necrosis may be seen but are considered rare (Olsen et al. 1989). In situations of persistent injury, tubular atrophy and interstitial fibrosis develop leading to a chronic phase of renal function impairment (Adachi et al. 2014) characterized by a vicious circle of tubular atrophy, fibrosis, and glomerulosclerosis, ultimately causing end stage renal disease (Ferenbach and Bonventre 2015). In ATI, regeneration commences by proliferation of surviving dedifferentiated tubule cells that express markers of proliferation and cellular immaturity like Ki67 and Cluster of Differentiation 133 (CD133) respectively (Bussolati et al. 2005; Loverre et al. 2008; Kusaba et al. 2014). Whether these immature cells are consistently present (dormant) renal progenitor cells or dedifferentiated mature tubular cells is still disputed, but it is generally assumed that CD133 positive proliferating cells play an important role in regeneration (Kusaba et al. 2014; Lombardi et al. 2015).

Despite substantial knowledge about morphological changes and regenerative mechanisms in animal models, little is known about their association with recovery of renal function after ATI in humans. It has recently been shown that morphological ATI characteristics in post‐RTX biopsies taken at 6 weeks, and 3 and 6 months correlate with eGFR at the time of biopsy. Whether morphology can be used in prognostics is unknown, but might prove useful given the frequency of biopsies taken during episodes of DGF (mainly to rule out acute rejection) (Schumann‐Bischoff et al. 2018). It is of note that for transplant patients, validated histological scoring systems for assessing chronic damage based on the Banff criteria and clinical donor parameters do exist (Nyberg et al. 2003; De Vusser et al. 2013).

We hypothesize that morphological and immunohistochemical markers of damage and regeneration in patients with DGF due to ATI correlate with graft functional recovery and add prognostic value to existing clinical models. We therefore evaluated biopsies of patients with DGF after RTX with ATI and no other concurrent pathology for morphological characteristics of damage and immunohistochemical characteristics of regeneration. These markers were examined in the renal cortical tissue as this is the main target of renal biopsies. A model that correlates morphological and immunohistochemical markers with renal outcome will not only provide a powerful prognostic tool, but might also provide insights into the mechanisms involved in renal injury and regeneration in humans.

## Materials and Methods

### Renal transplant patients

We retrospectively evaluated all patients aged over 18 who received a cadaveric kidney transplant (Kootstra et al. 1995) in the UMC Utrecht and UZ Leuven between 2005 and 2014, or those who received a cadaveric kidney transplant or a living related kidney transplant in the AMC Amsterdam between 2002 and 2014. We included patients who underwent indication biopsies taken 5–9 days after RTX because of DGF defined as the need for dialysis in the first week posttransplantation, or because of slower than expected recovery of function in the living donor group. We excluded patients with DGF biopsies showing concurrent acute pathology other than ATI (e.g. acute rejection).

We obtained clinical data regarding recipient age, gender and cold ischemia time from patient records. In addition, the following characteristics were collected to compose a clinical donor score described by Nyberg et al. (2003): donor age, cause of death, endpoint eGFR, and HLA mismatches. Donor history of hypertension yielded too many missing data points (missing data 57%) to compose the full Nyberg score, therefore a modified version without history of hypertension was composed.

Renal function was expressed as estimated Glomerular Filtration Rate (eGFR) 3, 6, and 12 months after RTX. eGFR was calculated using the Chronic Kidney Disease Epidemiology Collaboration (CKD‐EPI) formula (Levey et al. 2009). Serum creatinine levels were assessed using colorimetric enzymatic assay (Beckman Coulter, Brea, CA).

### Histochemistry

An ultrasound‐guided 22‐mm biopsy was taken from the renal cortex using a 16‐Gauge needle. Renal biopsies were formalin‐fixed and paraffin‐embedded using standard procedures. For all staining procedures 3 *μ*m sections were cut, deparaffinised with xylene and rehydrated with a 100, 96, and 70% ethanol sequence. Once rehydrated, the sections were stained with periodic acid‐Schiff (PAS) and hematoxylin and eosin using standard procedures.

To quantify histological tubular damage and interobserver variability, PAS–stained slides were scored by three blinded and independent observers (T.T.P., L.L.F., S.F.) in the UMC Utrecht and Amsterdam UMC cohort. Given the low interobserver variation (see Results), 1 observer scored the Leuven cohort (T.T.P.). Tubular dilatation, tubulointerstitial edema, nonisometric cell vacuolization and casts were given a score between 0 and 4 as a percentage of the total cortical area of the biopsy (0 = 0–1%, 1 = >1–10%, 2 = >10–25%, 3 = >25–50%, and 4 > 50%). Tubular atrophy was quantified in a similar fashion.

### CD133 and Ki67 Immunohistochemistry

Biopsies in the UMCU cohort were available for further immunohistochemical evaluation of parameters of regeneration: proliferation marker Ki67 and progenitor cell marker CD133 (*n* = 25). Endogenous peroxidase was blocked by H_2_0_2_‐incubation followed by antigen retrieval by boiling in EDTA pH9 or Citrate pH6 followed by overnight incubation at 4°C with mouse anti‐CD133 (1:10; AC133, Miltenyi, Bergisch‐Gladbach, Germany), or 1 h at room temperature with rabbit anti‐Ki67 (1:200; SP6, Thermo‐Fisher, Waltham, USA). After thorough rinsing with PBS, the sections were incubated with Brightvision Alkaline‐Phosphatase linked secondary antibodies (Immunologic, Duiven,the Netherlands) according to initial primary antibody species. For Ki67 and CD133 quantification, biopsies were blinded and ten high power fields per biopsy (200× magnification) were studied by two independent observers (T.T.P., L.L.F.). The number of immuno–positive tubular cells or nuclei were counted and averaged per biopsy.

### Ethics

This study was performed according to the declaration of Helsinki and Istanbul and the ethical guidelines of our institution. All clinical data and patients samples were anonymized. Anonymous use of redundant tissue for research purposes is part of the standard treatment agreement with patients in our hospitals. Additional ethical approval was therefore not required (van Diest 2002).

### Statistics

Normality was assessed using P‐plots, histograms and the Shapiro–Wilk test. Correlations between continuous and/or ordinal variables were tested in univariate analysis using linear regression or one‐way ANOVA with linear contrasts. Interaction over time was assessed with mixed linear models. Relations between clinical parameters and biopsy characteristics were assessed with Principle Component Analysis (PCA). Multivariate analysis was performed using linear mixed models, the *R* ^2^‐value given is calculated as variance explained by the fixed effects only (Nakagawa and Schielzeth 2012). Receiver Operator Characteristic (ROC) curve analysis was used to analyze discriminative ability. Missing eGFR‐values were imputed using the “last and next” method, averaging the previous and following observation when they were no more than 9 months apart (Engels and Diehr 2003). Missing values, with exception of the outcome variable eGFR, were imputed using multiple imputation (*M* = 5, 10 iterations per database, combined with Rubin's rule). *P*‐values below 0.05 were considered statistically significant. Statistical analysis was performed with SPSS for Windows version 20.0.0. Interobserver variation was tested with Kendall's coefficient of concordance with statistical program R for McIntosh version 3.3.

## Results

### Clinical characteristics and renal function after DGF due to ATI

We identified 94 patients with DGF due to ATI without concurrent acute pathology. All patients received a combination of (methyl)predniso(lo)ne, tacrolimus and mycophenolic acid. In addition, 50 patients received induction therapy with the monoclonal anti‐IL2 receptor antibody Basiliximab (Simulect™). Baseline characteristics in relation to renal function are shown in Table 1. A modified version of the Nyberg score, a well validated clinical score for cadaveric grafts, correlated with renal function at 1 year in patients who received a cadaveric graft (Nyberg et al. 2003). Of the separate clinical characteristics, donor age, donor eGFR, and the number of HLA mismatches were significantly associated with eGFR at 1 year.

**Table 1:** Association between eGFR at 1 year and clinical characteristics of kidney transplant recipients*

Characteristic		eGFR 1 year	
		Correlation‐ coefficient	*P*‐value¶
Patients (*N*)	94	–	–
Donor type (Heart beating/nonHeart beating/Living)	32/56/6	–	0.160
Nyberg score†	17.6 ± 7.4	**−0.42**	**<0.0005**
Donor age (years)	52.0 ± 14.0	**−0.41**	**<0.0005**
Cause of death (CVA/other/missing)†	42/44/2	**−**0.20	0.09
Donor eGFR (mL/min/1.73 m^2^)	89.2 ± 31.0	**0.29**	**0.02**
HLA mismatches (*N*)	2.5 ± 1.6	**−0.24**	**0.03**
Donor gender (m/f/missing)	46/34/14	0.04	0.73
Cold ischemia time (h)	16.6 ± 6.1	**−**0.13	0.25
Recipient age (years)	55.3 ± 12.8	**−**0.04	0.69
Recipient gender (m/f)	53/41	0.05	0.65
Basiliximab induction (yes/no)	51/43	**−**0.06	0.63

* Values are shown as means ± SD or number of patients.

¶ *P*‐values were obtained by linear regression. Significant values are highlighted. *P*‐values <0.05 were considered significant.

† Only applicable to cadaveric donors.

### Morphological characteristics of damage are reproducible and correlate to renal function within the first year after transplantation

**Figure 1:**
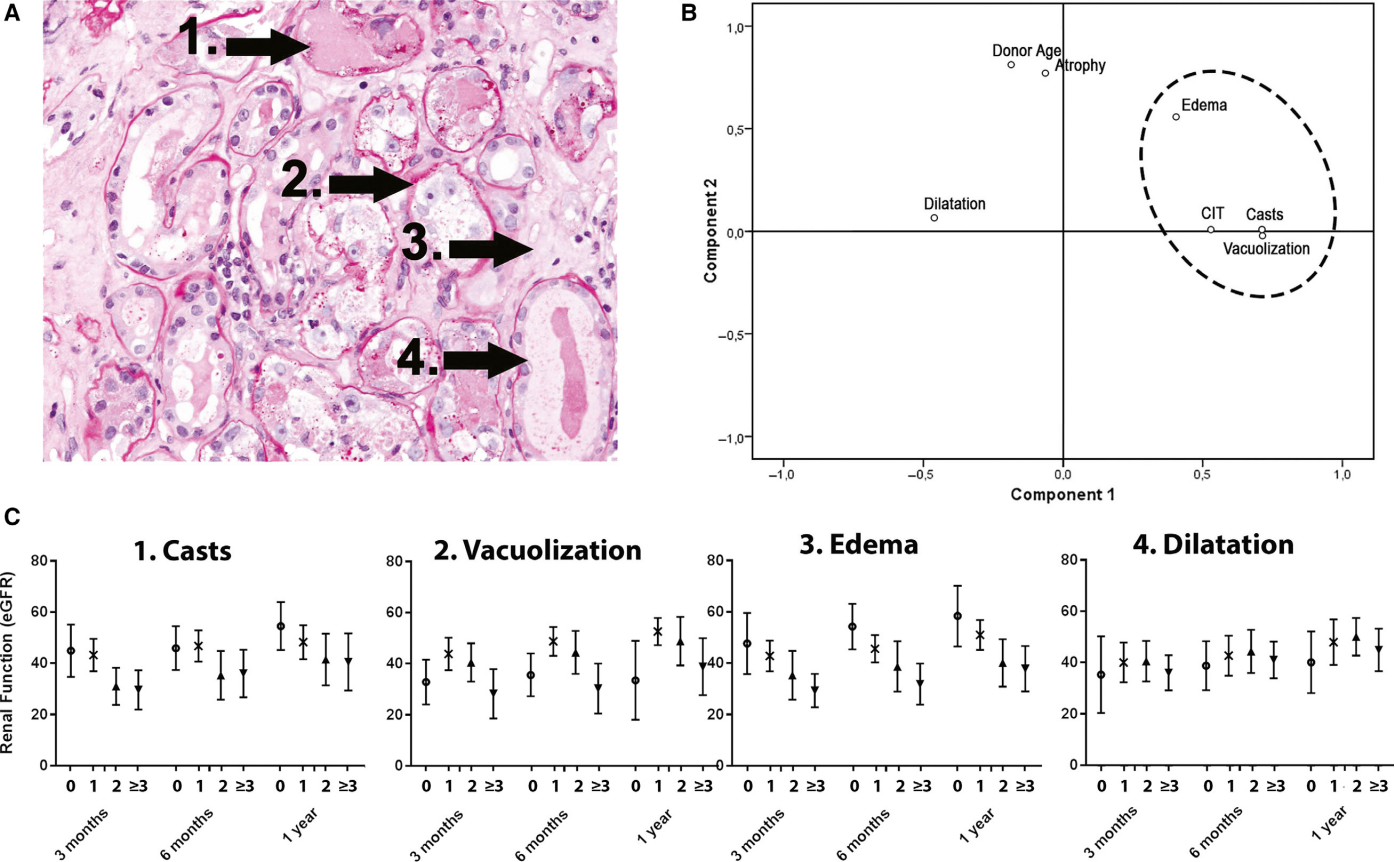
Histological damage correlates to renal function at 1 year. (A) Representative PAS stained micrograph of renal cortex with extensive tubular damage, displaying (1) Casts, (2) Vacuolization, (3) Edema, and (4) Dilatation. (B) Principal Component Analysis of morphological characteristics of acute (dilatation, edema, vacuolization, casts) and chronic (tubular atrophy) tubular injury, and clinical parameters associated with acute (CIT = Cold Ischemia Time) and chronic (donor age) tubular injury. (C) Association between severity of morphological parameters of acute tubular injury (percentage of cortex afflicted: 0–1% = **○**, >1–10% = *x*, >10–25% = ■, >25% = ▲) and eGFR at 3, 6, and 12 months after transplantation (mean 95% CI).

In order to analyze the relationship between the histological characteristics of ATI and renal function in the first year after transplantation, readily observable morphological characteristics of ATI (dilatation, vacuolization, casts, and edema) were scored (Table 2, Fig. 1). The concordance of all characteristics scored by three independent observers in the Utrecht and Amsterdam cohort (*n* = 57, Table 3) determined using Kendall's coefficient of concordance, ranged from moderate (*W* = 0.57 for edema) to excellent (*W* = 0.85 for casts).

**Table 2:** Correlations between renal outcome at 3 months, 6 months, and 1 year and morphological damage characteristics*

Characteristic	Median [range]¶	eGFR 3 months		eGFR 6 months		eGFR 1 year		Interaction with time
		*η* ^2^	*P*‐value†	*η* ^2^	*P*‐value†	*η* ^2^	*P*‐value†	*P*‐value‡
Damage combined	6 [1–13]	0.184	**<0.0005**	0.176	**<0.0005**	0.130	**<0.0005**	0.38
Dilatation**	2 [0–4]	0.001	0.77	<0.001	0.88	0.001	0.75	0.53
Vacuolization**	1 [0–5]	0.041	0.06	0.057	**0.02**	0.012	0.30	0.10
Casts**	1 [0–4]	0.101	**0.003**	0.049	**0.04**	0.061	**0.03**	0.41
Edema**	1 [0–4]	0.127	**<0.001**	0.182	**<0.0005**	0.142	**0.001**	0.24

* Values are shown as medians [range].

¶ Values were averaged from observations of three observers in the Amsterdam cohort and one observer in the Leuven cohort.

† Obtained by one‐way ANOVA using linear contrasts to investigate linear relation between variables. *η* ^2^ represents variance in the outcome explained and is comparable to the *R* ^2^‐value. Significant values are highlighted.

‡ Assessed for interaction with linear mixed model to investigate whether there was a significant interaction with time. *P*‐values <0.05 were considered significant.

‡ Assessed for interaction with linear mixed model to investigate whether there was a significant interaction with time. *P*‐values <0.05 were considered significant.

** Percentage of cortex affected 0–1%: 0; >1–10%:1; >10–25%:2; >25–50%:3; >50%:4.

**Table 3:** Interobserver variation*

Characteristic	W
Dilatation†	0.62
Vacuolization†	0.61
Casts†	0.85
Edema†	0.57

* Interobserver agreement in the Amsterdam and Utrecht cohort between three independent observers was calculated using Kendall's coefficient of concordance; 0 represents no agreement and 1 represents perfect agreement.

† Percentage of cortex affected: 0–1%: 0; >1–10%:1; >10–25%:2; >25–50%:3; >50%:4.

Next, we assessed the relation between individual histological parameters and renal function. Edema and casts displayed a significant linear relation with renal function at all three time‐points (Table 2, Fig. 1C). Linear mixed model analysis revealed that the effect on renal function from casts and edema was present from 3 months after transplantation and remained constant between 3 months and 1 year after transplantation (Table 2). We used Principle Component Analysis to isolate whether these morphological characteristics were associated with acute or chronic kidney damage. The rotating plot showed distinct grouping of cold ischemia time, edema, casts, and vacuolization, whilst the tubular atrophy grouped with donor age. Tubular dilatation was associated with neither groups (Fig. 1B).

### Markers of regeneration do not correlate to renal function within the first year, even in the context of damage

**Figure 2:**
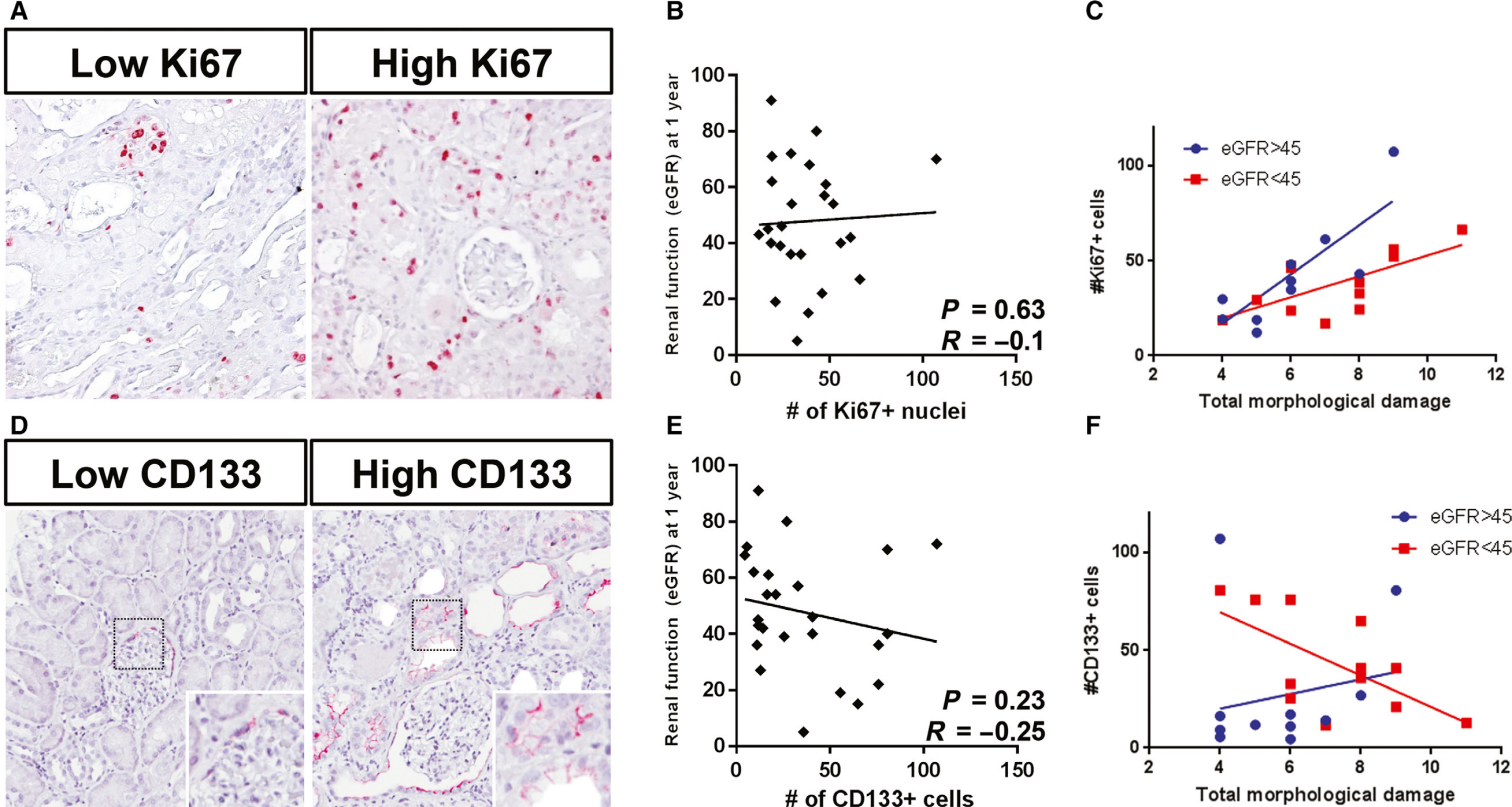
: Markers of proliferation and regeneration do not associate with renal recovery after DGF and do not show distinct clusters of favorable outcome in the context of histological damage. (A) Representative micrographs of low and high numbers of Ki67 +  tubular cells. (B) Pearson correlation between 6‐month eGFR and the number of Ki67 +  tubular cells. (C) Scatter plot of # of Ki67 +  nuclei and total morphological damage (Dilatation + Vacuolization + Casts + Edema) with distinct regression lines for best (eGFR > 45 mL/min, blue open circles) and worst (eGFR < 45 mL/min, red crosses) renal outcomes. (D) Representative micrographs of low and high numbers of CD133 +  tubular cells. (E) Pearson correlation between 6‐month eGFR and the number of CD133 +  tubular cells. (F) Scatter plot of # of CD133 +  cells and total morphological damage (Dilatation + Vacuolization + Casts + Edema) with distinct regression lines for best (eGFR > 45 mL/min, blue open circles) and worst (eGFR < 45 mL/min, red crosses) renal outcomes.

To assess the regenerative response, we quantified the number of Ki67+ tubular nuclei and CD133+ tubular cells (Fig. 2A and D; Table 4). The Ki67 antigen is expressed in cells during all active phases of the cell cycle, and is used as a marker for cells in proliferation (Jurikova et al. 2016). CD133 is an epitope found on the glycoprotein Prominin‐1 and is expressed by a wide variety of stem cells (Shmelkov et al. 2005). We did not find a significant correlation between renal function at 3, 6 and 12 months and either tubular Ki67+ cells (Fig. 2B; Table 4) or tubular CD133+ cells (Fig. 2E; Table 4), or both combined using standardized *Z*‐scores (Table 4).

**Table 4:** Correlations between renal outcome at 3 months, 6 months, and 1 year and morphological damage characteristics and regenerative characteristics*

Characteristic	Mean ± SD	eGFR 3 months		eGFR 6 months		eGFR 1 year		Interaction over time
		*r*	*P*‐value†	*r*	*P*‐value†	*r*	*P*‐value†	*P*‐value‡
Regeneration combined (standardized)**	–	−0.22	0.28	−0.23	0.27	−0.10	0.63	0.49
CD133¶	35.5 ± 29.4	−0.30	0.14	−0.25	0.63	−0.20	0.33	0.84
Ki67¶	37.4 ± 20.8	−0.04	0.85	−0.10	0.63	0.05	0.83	0.34

* Values are shown as means ± SD.

¶ Number of immunopositive cells per high–power field. Values were averaged from observations of two observers in the Utrecht cohort.

† Obtained by linear regression. Significant values are highlighted.

‡ Assessed for interaction with linear mixed model to investigate whether there was a significant interaction with time.

** Combined score made with standardized *Z*‐scores to account for different means and standard deviations.

The extent of damage might confound the correlation between parameters of regeneration and renal function. Insufficient regeneration as response to severe injury might worsen outcome; patients with good outcome are however expected to have an appropriate regenerative response to the amount of injury. We therefore used an interaction term to compare the regression coefficients of parameters of renal damage and regeneration (CD133 and Ki67) in patients with good outcome (eGFR ≥ 45 mL/min/1.73 m^2^ at 1 year [*n* = 13]) versus recipients with bad outcome (eGFR < 45 mL/min/1.73 m^2^ at 1 year [*n* = 12]) (total damage: Fig. 2C and F; edema + casts only: Fig. S1). Regression analysis of the regenerative parameters with the morphological characteristics as dependent variable showed similar regression slopes in both outcome groups for Ki67 (*B* = 0.05 vs. *B* = 0.04, *P* = 0.743) and CD133 (*B* = 0.02 vs. *B* = −0.03, *P* = 0.125). It is of note that the number of Ki67+ tubular cells correlated significantly with total damage regardless of renal function (*R* = 0.51, *P* = 0.01). Thus, neither proliferation marker Ki67 nor stem cell marker CD133 correlated with renal outcome, even when assessed in the context of histological damage.

### Morphological parameters of damage are associated with renal function independently of clinical characteristics

To explore the independent effect of the morphological damage characteristics on renal function, we performed linear mixed model analysis of the injury markers and clinical characteristics known to be associated with eGFR after transplantation (donor age, last known donor eGFR, HLA mismatches) (Nyberg et al. 2003). Linear mixed model analysis revealed that edema and casts were independently associated with eGFR within the first year after RTX (Table 5). The discriminant function from the linear mixed model (4*edema + 3*casts + 0.5* donor age) significantly discriminated between patients that did (*N* = 73) and did not (*N* = 21) reach an eGFR above 30 mL/min/1.73 m^2^ within the first year after transplantation (AUC = 0.819, *P* < 0.0005). It is of note that the Nyberg score is a model using clinical parameters to predict eGFR in patients who received a kidney from deceased donors. In the subgroup of our cohort that received a kidney from a deceased donor (*n* = 88), the model including donor age, casts and edema predicted eGFR within the first year (*R* ^2^ = 0.29, *P* < 0.0005) better than the Nyberg score (*R* ^2^ = 0.14, *P* < 0.0005).

**Table 5:** Estimates of fixed effects of linear mixed model of the association between morphological characteristics, clinical characteristics, and outcome variable eGFR within the first year after transplantation

Variables	Univariate analysis		Multivariate analysis	
	Estimate (B)	*P*‐value*	Estimate(B)	*P*‐value*
Clinical characteristics				
Donor age	−0.57	**<0.0005**	−0.44	**<0.01**
HLA mismatches	−3.05	**<0.01**	−1.27	0.27
Donor eGFR	0.17	**0.01**	0.05	0.82
Biopsy characteristics¶				
Dilatation	0.28	0.89	–	–
Vacuolization	−3.24	0.08	−0.75	0.66
Casts	−4.38	**<0.0005**	−3.19	**<0.05**
Edema	−6.15	**<0.0005**	−4.29	**<0.01**

* *P*‐values were obtained by linear mixed model analysis. Significant values are highlighted. *P*‐values <0.05 were considered significant.

¶ Percentage of cortex affected 0–1%: 0; >1–10%:1; >10–25%:2; >25–50%:3; >50%:4.

## Discussion

This multi–center retrospective cohort study represents the largest cohort to date that examines the morphological characteristics of ATI *during* DGF after RTX. We show that a subset of histological characteristics of ATI significantly correlates to renal functional recovery in RTX patients with DGF and improves the predictive value of traditional clinical parameters in our cohort. Analysis of histological parameters traditionally associated with ATI revealed that tubulointerstitial edema and luminal casts significantly related to renal function at 3, 6 and 12 months after transplantation. Moreover, a predictive model that combines donor age with histological parameters edema and luminal casts, outperformed a widely–validated clinical score in predicting eGFR within the first year after transplantation. Interestingly, we show that parameters of tubular regeneration and proliferation did not correlate to renal functional recovery, even when assessed in the context of tubular damage.

The low interobserver variability of histological parameters investigated is essential for potential clinical application as a prognostic tool. Observers working closely together may influence each other's scoring (Furness et al. 2003). In our study, evaluation of the histological parameters showed an overall good reproducibility, also when compared with a pathologist from an independent center (S.F.). The interobserver variability was comparable to well–known histological scoring models such as the Oxford classification of IgA nephropathy and the ISN/RPS2003 classification of Lupus Nephritis (ICC 0.46–0.78, ICC 0.41 respectively) (Grootscholten et al. 2008; Roberts et al. 2009). Another study found high interobserver variability for casts in protocol and indication biopsies after RTX as opposed to the low interobserver variability we observed (Schumann‐Bischoff et al. 2018). This discrepancy can be explained by the difference in underlying disease severity, since most of these biopsies were not taken in patients with acute kidney injury.

Our study is the first that shows an association between standardized and reproducible biopsy characteristics of ATI during DGF and renal function at 3, 6, and 12 months after RTX. Other publications about the association between morphological characteristics of ATI after RTX and renal function vary widely in their results and the morphological characteristics that are quantified. Recently, it was demonstrated that accumulation of characteristics of ATI in indication and protocol biopsies of transplant patients is associated with eGFR at the time of the latest biopsy (Schumann‐Bischoff et al. (2018). Another study demonstrated an inverse relation between renal function at 1 year and the mere presence of ATI (defined as presence of epithelial swelling, loss of brush border, dilatation, or cytoplasmic vacuolization) in protocol or indication biopsies taken between 2 and 6 months after transplantation (Gwinner et al. 2008). However, two studies failed to show a relation between the presence or degree of ATI, defined by apical blebbing, cell sloughing, and vacuolization, in *pre*transplantation biopsies and renal outcome (Oppong et al. 2016) (Hall et al. 2014). The difference between these studies and ours is most likely explained by quantification of different morphological aspects, quantification on wedge biopsies versus needle biopsies, and the timing of biopsy. Finally in a small study of non‐RTX related ATI, Abdulkader et al. (2008) showed that a combination of acute and chronic damage (ATI, interstitial fibrosis, tubular atrophy, and interstitial infiltrate) in biopsies of native kidneys related to renal function.

The associations between morphological characteristics and outcome found in this study are in line with pathophysiological insights from animal and human studies. Studies in mice show that severe ATI due to prolonged ischemia time increases the amount of structural damage represented by the amount of intraluminal debris and an increase in tubular backleak, a main determinant of tubulointerstitial edema (Donohoe et al. 1978). The PCA plot in our study suggests that the same mechanism of prolonged ischemia time is indeed associated with the severity of ATI in humans, represented by the amount of casts, interstitial edema, and vacuolization. The unexpected quadratic relationship observed between vacuolization and eGFR might be explained by the role of autophagy in ATI, which can serve as a mechanisms of cell death but has also been shown to be protective (Suzuki et al. 2008; Jiang et al. 2012). Abundant and widespread vacuolization may therefore represent extensively damaged tubules, whilst absence of vacuolization might indicate an impaired autophagy response to damage. In animal studies, the degree of interstitial edema has been associated with severity of disease and worse recovery, which is attributed to increased intrarenal pressure generated by the noncompliant renal capsule (Herrler et al. 2010; Hueper et al. 2013; Tewes et al. 2017). An increase in intrarenal pressure further hampers perfusion and filtration due to a diminished transcapillary pressure difference, which is associated with prolonged ATI in humans (Ramaswamy et al. 2002).

Surprisingly, despite the importance of regeneration in the recovery of renal function, parameters of regeneration did not relate to renal outcome, even when corrected for the extent of damage. Proliferative activity however did correlate with damage, as has been observed previously (Pizov and Friedlaender 2002). The concept of maladaptive repair might explain this finding. Upon injury, surviving tubular cells initiate proliferation but may get arrested in the G2/M phase of the cell cycle, acquiring a profibrotic and proinflammatory phenotype, excreting factors negatively affecting outcome like for example CTGF (Yang et al. 2010; Falke et al. 2014; Thomasova and Anders 2015). CD133 and ki67 might therefore not only mark active cycling regenerating cells but also arrested, profibrotic tubular cells; consequently their presence does not necessarily represent favorable regenerative potential or activity. In addition, we cannot exclude that other factors like drug toxicity, or capillary rarefaction obscured a possible correlation of regenerative parameters and renal function (Rosen and Stillman 2008; Ferenbach and Bonventre 2015).

In this study we identified the histological parameters of ATI in DGF biopsies that correlate to renal outcome and improve the predictive value of validated clinical score. Besides confirming the pathophysiological insight derived from animal models in humans, our findings can have clinical consequences. Histological scoring can improve prognostication during ATI after RTX and can be used to evaluate the effect of peri transplantation interventions such as machine‐perfusion of donor organs or specific medical treatments (Aufhauser et al. 2016; Hosgood et al. 2016; Jochmans et al. 2016). Although our study was limited to post‐RTX ATI, the parameters used are universal tubular damage markers and our findings might also apply to non‐RTX associated ATI. This is supported by the aforementioned findings of Abdulkader et al. (2008). Finally, the observation that parameters derived from animal and preclinical models of regeneration did not correlate to renal outcome might relate to maladaptive regeneration processes which deserves further investigation.

## Conflict of Interest

The authors declare no conflicts of interest.

## Supporting information




**Figure S1.** (A) Scatter plot of # of Ki67 + nuclei and sum of morphological characteristics that correlate with eGFR in follow‐up(Casts+Edema) with distinct regression lines for best (eGFR > 45 mL/min, blue open circles) and worst (eGFR < 45 mL/min, red crosses) renal outcomes. (B) Scatter plot of # of CD133 + nuclei and sum of morphological characteristics that correlate with eGFR in follow‐up(Casts+Edema) with distinct regression lines for best (eGFR > 45 mL/min, blue open circles) and worst (eGFR < 45 mL/min, red crosses) renal outcomes.
