# Supplementary figures and images for: Histological characteristics of Acute Tubular Injury during Delayed Graft Function predict renal function after renal transplantation
Source: Physiol Rep. 2019 Feb 28;7(5):e14000. doi: 10.14814/phy2.14000 (PMC6395310; doi:10.14814/phy2.14000)

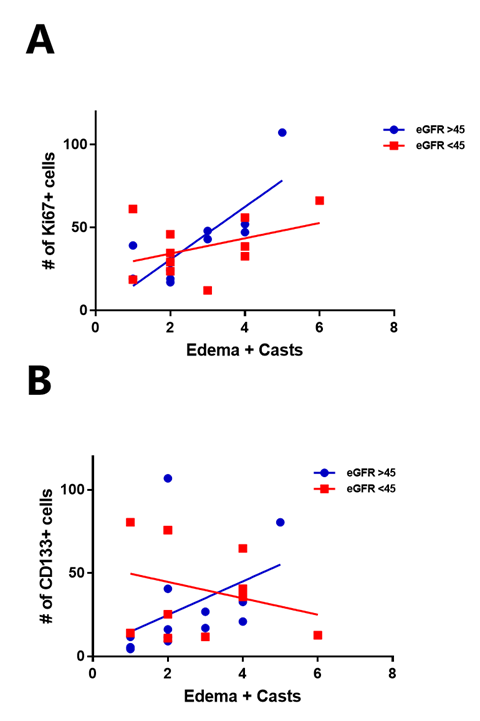

Supplement: Supplementary file 1 — Figure S1. (A) Scatter plot of # of Ki67 + nuclei and sum of morphological characteristics that correlate with eGFR in follow‐up(Casts+Edema) with distinct regression lines for best (eGFR > 45 mL/min, blue open circles) and worst (eGFR < 45 mL/min, red crosses) renal outcomes. (B) Scatter plot of # of CD133 + nuclei and sum of morphological characteristics that correlate with eGFR in follow‐up(Casts+Edema) with distinct regression lines for best (eGFR > 45 mL/min, blue open circles) and worst (eGFR < 45 mL/min, red crosses) renal outcomes. [file PHY2-7-e14000-s001.tif]
